# The therapeutic challenge of giant splenic artery aneurysm: a case repport

**DOI:** 10.1590/S1679-45082017RC3873

**Published:** 2017

**Authors:** Paulo Kauffman, Antonio Luiz de Vasconcellos Macedo, Roberto Sacilotto, Adriano Tachibana, Sergio Kuzniec, Lucas Lembrança Pinheiro, Nelson Wolosker

**Affiliations:** 1 Hospital das Clínicas, Faculdade de Medicina, Universidade de São Paulo, São Paulo, SP, Brazil.; 2 Hospital Israelita Albert Einstein, São Paulo, SP, Brazil.; 3 Hospital do Servidor Público Estadual “Francisco Morato de Oliveira”, São Paulo, SP, Brazil.

**Keywords:** Aneurysm/diagnosis, Aneurysm/therapy, Splenic artery/pathology, Case reports, Aneurisma/diagnóstico, Aneurisma/terapia, Artéria esplênica/ patologia, Relatos de casos

## Abstract

Giant splenic artery aneurysm is a rare condition that represents an eminent life threatening for the patient, requiring, therefore, urgent surgical correction. A 61-year-old woman, former smoker, hypertensive, hypercholesterolemic and multipara sought our service because of a large tumor in the mesogastrium, which was an abdominal ultrasound finding. Despite the size of the tumor, the patient was asymptomatic. The angiotomography and the magnetic resonance image of the abdomen were suggestive of giant splenic artery aneurysm with more than 10cm in diameter that was confirmed by an angiography. She underwent surgery, open splenectomy, and partial aneurysmectomy. The approach of the celiac artery, which was ligated, was only possible with medialvisceral rotation because there was no possibility to view it through the anterior access. The histopathological test of aneurysmatic wall revealed atheroma plaques in the intima. The patient progressed without complications and she was discharged cured. In general, giant splenic artery aneurysms are symptomatic, however, as in the case we report, it may be asymptomatic and found in abdominal imaging exam. Although less invasive Interventional methods exist, such as laparoscopy and endovascular techniques, they were considered inappropriate in this case. Conventional open surgery should be the therapy of choice for a giant splenic artery aneurysm.

## RESUMO

O aneurisma gigante da artéria esplênica constitui condição rara, que representa risco de vida iminente para o paciente, necessitando, consequentemente, de correção cirúrgica urgente. Mulher de 61 anos, ex-fumante, hipertensa, com hipercolesterolêmica e multípara nos procurou por apresentar grande tumor no mesogástrio, achado de ultrassonografia abdominal. Apesar das dimensões do tumor, era assintomática. Angiotomografia e ressonância magnética de abdômen sugeriam tratar-se de aneurisma gigante de artéria esplênica com mais de 10cm de diâmetro, confirmado por angiografia. Foi submetida a tratamento cirúrgico aberto, tendo sido realizadas esplenectomia e aneurismectomia parcial. A abordagem do tronco celíaco, que foi ligado, só foi possível com rotação visceral medial, pois não havia possibilidade de visualizá-lo pela via anterior. O exame anatomopatológico da parede do saco aneurismático revelou placas de ateroma na íntima. A paciente evoluiu sem intercorrências e teve alta hospitalar curada. Aneurismas da artéria esplênica de dimensões avantajadas, em geral, são sintomáticos, porém, como no caso em questão, podem ser assintomáticos e descobertos em exame de imagem do abdômen. Apesar de existirem métodos intervencionistas menos invasivos, como laparoscopia e técnicas endovasculares, eles não foram considerados adequados neste caso. Diante de um aneurisma gigante de artéria esplênica, a conduta terapêutica de eleição é a cirurgia convencional aberta.

## INTRODUCTION

Splenic aneurysm with more than 5cm diameter is rarely seen. The giant splenic artery aneurysm, greater than 10cm, is even rare. In 2005 there were only 12 cases of true splenic artery aneurysms described in the literature with such dimensions.^(^ ^1^ ^)^ Such case requires a careful plan and execution because of its intimal relation with surrounding abdominal organs. In Brazil, no cases of this disease have been reported so far.

We report a case of giant splenic artery aneurysm, highlighting technique and surgical approach employed.

## CASE REPORT

This was a 61-year-old woman without complains who was referred by her vascular surgeon after a routine abdominal ultrasonography that demonstrated a large proportion tumor in the mesogastric area. The patient had blood hypertension, which was controlled with 40mg furosemide taken once a day, hypercholesterolemia treated with 10mg rosuvastatin daily. There was a history of three previous gestations and she had smoked until the age of 19-year-old.

**Figure 1:**
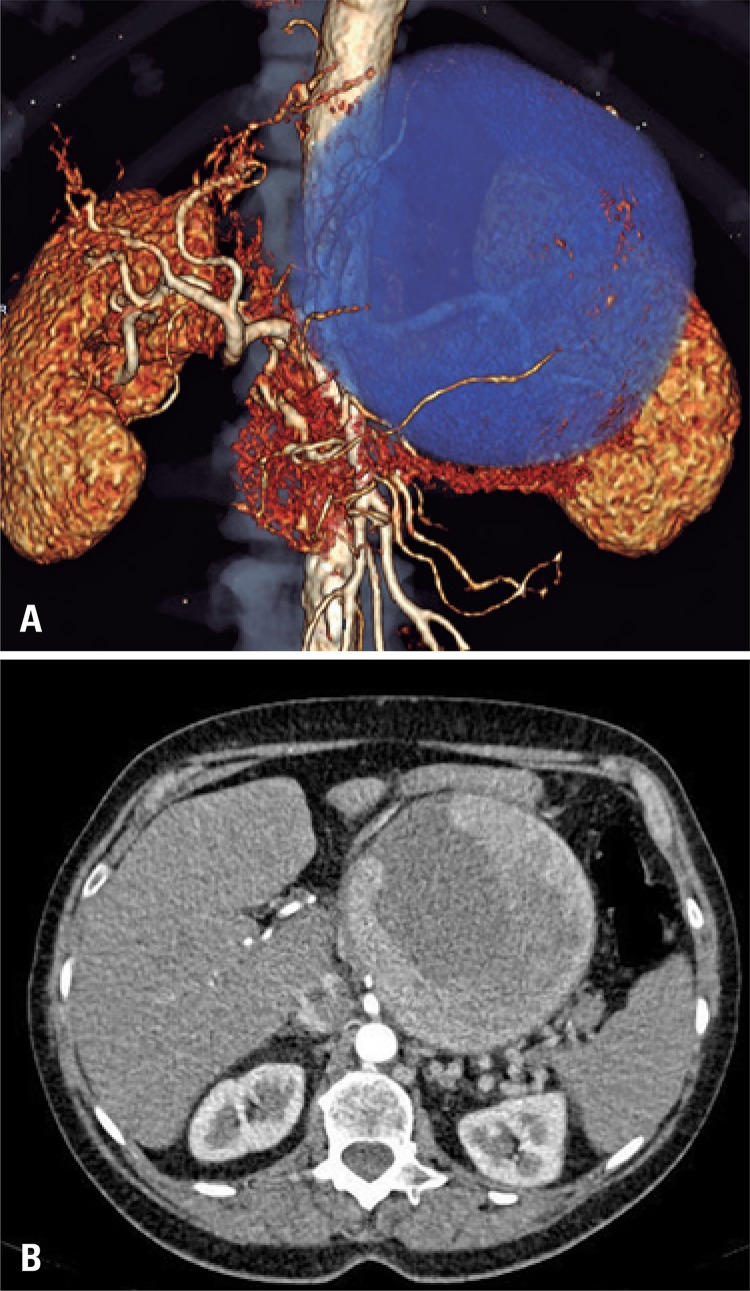
(A) Tridimensional reconstruction of the aneurysm. (B) Cross-sectional cutting

**Figure 2:**
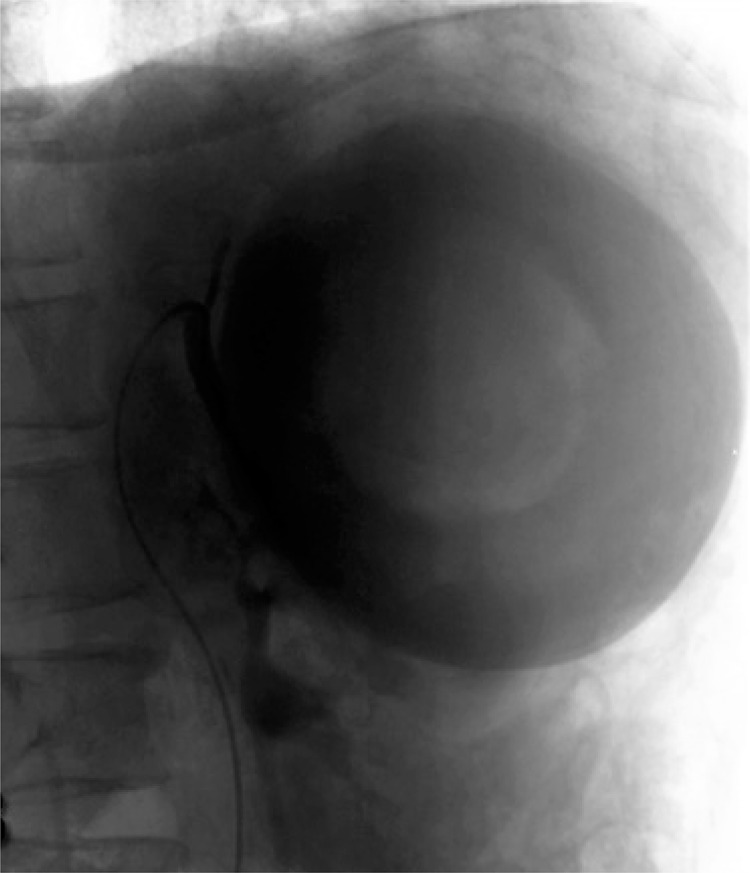
Selective arteriography

Upon physical examination, she reported pain on palpation in the upper left quadrant of abdomen. The patient underwent abdominal computed tomography without contrast agent that identified a giant splenic artery aneurysm measuring 11x10cm (Figures 1A and B). An intraoperative angiography confirmed the initial suspicion (Figure 2).

**Figure 3:**
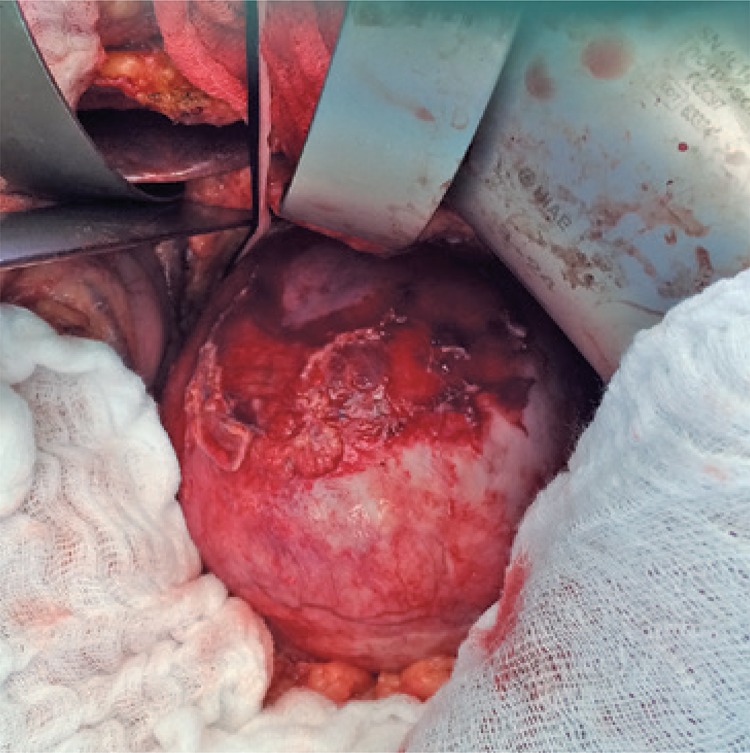
Intraoperative exposure of the aneurysm

Patient underwent surgical treatment by median xyphopubic laparotomy. The opened small omentum did not enable to approach celiac artery by epiplon retrocavity because aneurysm do not enabled its visualization. A dissection, therefore, was carried out resealing partly the right lateral wall of aneurysmatic sac with the aim to identify celiac artery that also enable its visualization. We opted to perform a medial visceral rotation with descending colon mobilization by the incision of lateral reflection of peritoneum, cranially prolonged with section of phrenicocolic and splenorenal ligament. The plan was achieved between pancreas and Gerota’s fascia, promoting anteromedial rotation of the spleen, pancreas and stomach, therefore identifying the tumors (Figure 3).

Left lobe of the liver was mobilized and retracted, sectioning the triangle liking, whereas gonadal vein, ureter, left kidney, left renal vein and adrenal vein remained *in situ*. During mobilization of spleen a laceration of its capsule occurred, which determined the splenectomy. The Omni-Tract retractor was placed to maintain the viscera to right, left pile of diaphragm was partial sectioned and suprarenal aorta dissected with identification of higher mesenteric artery and initial short segment of celiac artery that was initially clamped, verifying the persistence of pulsability in liver artery; a ligature of celiac artery was performed.

The aneurysm had a sac aspect and it affected splenic artery in its proximal and medium segments. After the opening of aneurysmatic sac, we observed retrograde bleeding of two orifices of pancreatic branches that were sutured and its anterior wall was resected, leaving intact the remain aneurysm that was adhered to pancreas.

The anatomopathological exam of the aneurysm wall showed atheroma plaque in the intimal and hyalinization of medium tunic, in addition to aggregated of compatible fibrin with mural trunk without malignant signs. The patient evolved without intercurrences and remained asymptomatic 6 months after surgical intervention.

## DISCUSSION

Splenic artery aneurysm can be true or pseudoaneurysms.^(^ ^2^ ^)^ The majority of aneurysms is true, and seen among women and presents as important risk factors the astherosclerosis, arterial hypertension, and multiparity – all these factors are described in our case. About 10% of patients with giant arterial aneurysm described in the literature had hepatic cirrhosis and 2.5% portal hypertension^(^ ^3^ ^)^ such conditions possible constitutes predisposing factors and they were not seen in our patient. In splenic artery pseudoaneurysm the main risk factor is pancreatitis in which pancreatic enzimes can promove desistegration of the arterial wall and, in case of pseudocist, it can establish a fistulae between pancreatitis and the artery^(^ ^2^ ^)^ – initially suggested in the our case, based on size of the tumor with blood flow in its interior. This hypothesis was ignored because there was no previous history of pancreatic disease, and more accurate computed tomography exam of abdomen revealed a normal pancreas.

Most of splenic aneurysm is located in distal third of the artery and they are saccular, a fact not observed in our case, in which aneurysmatic dilatation, only saccular,^(^ ^3^ ^)^ evolved to proximal segments of vessels, which seems to characterize the giant aneurysms.^(^ ^1^ ^)^ Despite of enlarged dimensions, the patient was asymptomatic and aneurysm was found in routine ultrasonography exam of the abdomen, and these finding are compatible with other series.^(^ ^4^ ^)^

There are several treatment options for these aneurysms, depending on age, patient’s general conditions, artery site and dimensions of aneurysmatic sac. The progress of endovascular techniques and they good results have stimulate their employment in such cases, *i.e*., presenting little aggression and low morbidity.^(^ ^5^ ^,^ ^6^ ^)^ Embolization with moles or cyanoacrylate glue have been used successfully in less voluminous and short neck saccular aneurysm.^(^ ^6^ ^)^ However, in giant aneurysms, such as in our case, the use of large number of moles to exclude aneurysm become unviable, not only for the cost of the procedure, but also for high probability of not achieving total exclusion. In addition, infectious complications, splenic infarct, and intense inflammatory process have been described, and they constitute an inconvenience for employment of this technique in such circumstances.^(^ ^1^ ^,^ ^7^ ^)^

Other endovascular treatment option, in cases that aneurysm affect proximal and medial segments of splenic artery, comprises the implantation of endoprosthesis to preserve irrigation of spleen and promote exclusion of aneurysm. However, accentuated tortuosity of artery can avoid the implantation, although there are successful reports using this technique.^(^ ^8^ ^)^ In our case, implantation of endoprosthesis was not considered because there was no proximal neck of splenic artery for endoprosthesis fixation.

The ultrasound-guided thrombin injection is another feasible option.^(^ ^9^ ^)^ However, this type of injection was disregarded to our patient because it was large size true aneurysm that massive thrombosis could cause complications.

Although endovascular techniques are highly developed, conventional open surgical treatment remains the gold-standard to approach splenic artery aneurysms^(^ ^10^ ^)^ mainly giant aneurysms such as the one reported in this case. Patients with intact aneurysm have low morbidity and mortality, and complete resolution with no need of later control. The open approach is complex and constitutes a true challenge to the surgical team, because of the impossibility to approach celiac artery by an anterior route due to the presence of large putative mass. Our case resemble the one reported by Yadav et al.,^(^ ^10^ ^)^ in which the patient had good general condition, lack of pancreatic disease, large size aneurysm, and who underwent open surgical treatment. However, distal aneurysm site in artery and decision of totally resection led Yaday and colleagues to perform an aneurysmectomy associated with splenectomy and caudal pancreatectomy, which constitutes a more aggressive surgical intervention than the one used in our case. We used only a partial aneurysmectomy with presentation of aneurysmatic sac wall adhered to pancreas – the approach adopted by Pescarus et al.,^(^ ^1^ ^)^ in a similar case.

Whenever possible the spleen should be maintained. In proximal aneurysms, the resection and primary anastomosis are possibilities. In our patient the absence of proximal neck avoided arterial recovery.

## CONCLUSION

Large visceral aneurysms and, particularly, splenic aneurysms promote distortions in their arteries of origin, in addition to adherences and compressions of surrounding organs, therefore causing difficult and/or turning impossible endovascular access, avoiding open surgical correction, which is the first choice for treatment of giant aneurysms.
